# Umbilical Cord Mesenchymal Stromal Cell-Derived Exosomes Rescue the Loss of Outer Hair Cells and Repair Cochlear Damage in Cisplatin-Injected Mice

**DOI:** 10.3390/ijms22136664

**Published:** 2021-06-22

**Authors:** Stella Chin-Shaw Tsai, Kuender D. Yang, Kuang-Hsi Chang, Frank Cheau-Feng Lin, Ruey-Hwang Chou, Min-Chih Li, Ching-Chang Cheng, Chien-Yu Kao, Chie-Pein Chen, Hung-Ching Lin, Yi-Chao Hsu

**Affiliations:** 1 Department of Otolaryngology, Tungs’ Taichung Metroharbor Hospital, Taichung 435, Taiwan; tsaistella111@gmail.com; 2 Rong Hsing Research Center for Translational Medicine, National Chung Hsing University, 145, Guoguang Rd., South Dist., Taichung City 402, Taiwan; 3 Department of Medical Research, Mackay Memorial Hospital, Taipei 104, Taiwan; yangkd@mmc.edu.tw (K.D.Y.); cpchen@mmh.org.tw (C.-P.C.); 4 Department of Otolaryngology, Mackay Memorial Hospital, New Taipei City 251, Taiwan; 5 Department of Medical Research, Tungs’ Taichung Metroharbor Hospital, Taichung 435, Taiwan; kuanghsichang@gmail.com; 6 Graduate Institute of Biomedical Sciences, China Medical University, Taichung 406, Taiwan; rhchou@mail.cmu.edu.tw; 7 General Education Center, Jen-Teh Junior College of Medicine, Nursing and Management, Miaoli 356, Taiwan; 8 Department of Thoracic Surgery, Chung Shan Medical University Hospital, Taichung 402, Taiwan; frnklin@gmail.com; 9 School of Medicine, Chung Shan Medical University, Taichung 402, Taiwan; 10 Center for Molecular Medicine, China Medical University Hospital, Taichung 406, Taiwan; 11 Department of Medical Laboratory and Biotechnology, Asia University, Taichung 413, Taiwan; 12 Institute of Biomedical Sciences, Mackay Medical College, New Taipei City 252, Taiwan; ytrewq82325@gmail.com; 13 Laboratory Animal Service Center, Office of Research and Development, China Medical University, Taichung 406, Taiwan; chengjzvm@gmail.com; 14 Medical and Pharmaceutical Industry Technology and Development Center, New Taipei City 248, Taiwan; kcy7427p@gmail.com; 15 Department of Audiology and Speech-Language Pathology, Mackay Medical College, New Taipei City 252, Taiwan; hclin59@mmc.edu.tw

**Keywords:** mesenchymal stromal cells, cisplatin, hair cells, inner ear, ototoxicity, extracellular vesicles, exosomes

## Abstract

Umbilical cord-derived mesenchymal stromal cells (UCMSCs) have potential applications in regenerative medicine. UCMSCs have been demonstrated to repair tissue damage in many inflammatory and degenerative diseases. We have previously shown that UCMSC exosomes reduce nerve injury-induced pain in rats. In this study, we characterized UCMSC exosomes using RNA sequencing and proteomic analyses and investigated their protective effects on cisplatin-induced hearing loss in mice. Two independent experiments were designed to investigate the protective effects on cisplatin-induced hearing loss in mice: (i) chronic intraperitoneal cisplatin administration (4 mg/kg) once per day for 5 consecutive days and intraperitoneal UCMSC exosome (1.2 μg/μL) injection at the same time point; and (ii) UCMSC exosome (1.2 μg/μL) injection through a round window niche 3 days after chronic cisplatin administration. Our data suggest that UCMSC exosomes exert protective effects in vivo. The post-traumatic administration of UCMSC exosomes significantly improved hearing loss and rescued the loss of cochlear hair cells in mice receiving chronic cisplatin injection. Neuropathological gene panel analyses further revealed the UCMSC exosomes treatment led to beneficial changes in the expression levels of many genes in the cochlear tissues of cisplatin-injected mice. In conclusion, UCMSC exosomes exerted protective effects in treating ototoxicity-induced hearing loss by promoting tissue remodeling and repair.

## 1. Introduction

Mesenchymal stromal cells (MSCs) have been demonstrated to rescue cell apoptosis and repair injury in different tissues. They can be derived from different tissues, such as bone marrow [1,2], skin tissue [3,4], adipose tissue [5], placenta [6,7], and umbilical cord (UC) [8]. Among these tissue sources, UC has the characteristics of newborn tissue and is a promising source of MSCs for regenerative medicine [8,9]. Using UC as a source of MSC has several prominent advantages, including a painless collection process, and the derived MSCs have faster self-renewal capacity. In addition, UC-MSCs have shown the ability to differentiate into the key cell types in the three germ layers, to migrate to inflamed tissue regions, to repair tissue damages, and to regulate immune response. Intravenous injection of human UC blood MSCs can significantly ameliorate hearing loss by increasing the number of spiral ganglion neurons (SGNs) [10]. Xu et al. transplanted UCMSCs into deaf minipigs at 1 week after noise exposure and demonstrated that UCMSCs rescued hearing loss in the deaf pigs [11]. In addition, adipose tissue-derived MSCs can help in hearing repair through the secretion of factors or exosomes (paracrine effects) [12]. Therefore, investigating whether MSC-derived exosomes benefit cisplatin-induced hearing loss in mice is of considerable research interest. UCMSCs have been demonstrated to rescue cell apoptosis and repair tissue damage in different disease conditions, such as severe traumatic brain injury [8] and ischemic brain injury [13], mainly through the secretion of extracellular vesicles (EVs) [14]. Exosomes are EVs with a size range of 30–160 nm. They contain multiple growth factors as well as miRNA and lncRNA molecules, and they exert modulatory effects on injured tissues [15]. Notably, UCMSC exosomes [14,16] have been demonstrated to be effective for treating inflammatory diseases [17,18,19], ischemia–reperfusion injury [20], and neurodegenerative diseases [21].

Sensorineural hearing loss (SNHL) results from the damage or loss of cochlear hair cells (HCs) or SGNs [22]. The risk factors that lead to the loss of inner ear HCs include exposure to loud noises, aging, genetic mutations, use of ototoxic drugs, and autoimmune diseases [23,24,25,26]. The chemotherapeutic drug cisplatin is one of the ototoxic drugs that cause many cancer patients to have the side effects of SNHL. Furthermore, cisplatin not only causes HC loss but also damages SGNs and results in the degeneration of auditory nerve fibers [26,27,28]. Therefore, we chose cisplatin-induced SNHL in mice as the experimental model in this study.

To date, no FDA-approved drug exists for treating hearing loss. Biological approaches, such as Atoh1 gene therapy [29,30], IGF1 growth factor therapy [31,32], and MSC therapy [33], have been demonstrated to rescue the loss of cochlear HCs and ameliorate hearing loss in deaf animals. However, it remains unclear whether MSC-derived exosomes can ameliorate SNHL. In the present study, we further attempt to explore the therapeutic values of UCMSC-derived exosomes into impaired adult mammalian cochlea after HC damage. To that end, we initially characterized UCMSC-derived exosomes and demonstrated their efficacy in treating cisplatin-induced hearing loss in mice. The results from our study are a proof of principle; both systemic and local injection of UCMSC-derived exosomes may be a useful therapeutic strategy to repair the damaged mouse cochlea.

## 2. Results

### 2.1. Characterization of UCMSCs

**Figure 1:**
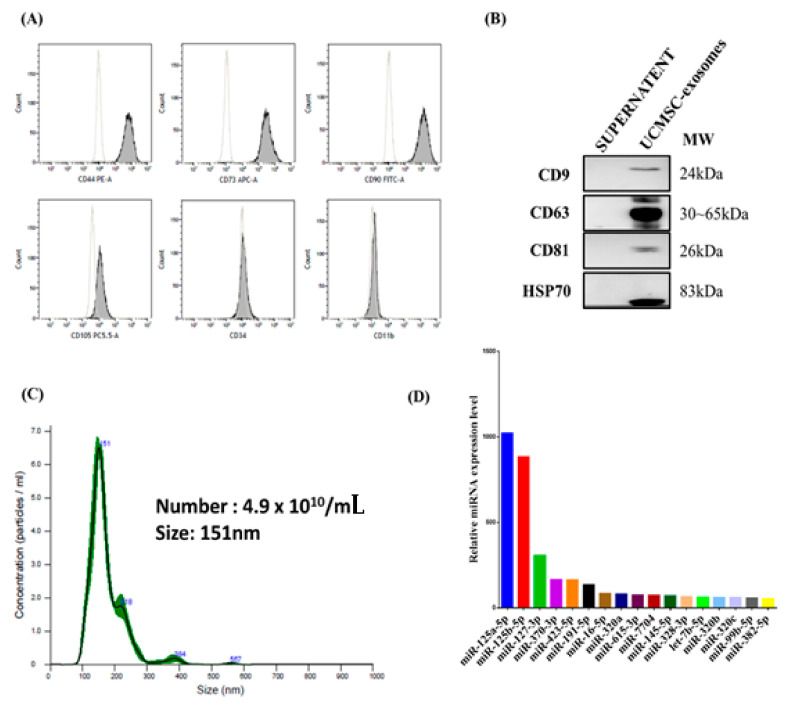
Isolation and characterization of human umbilical cord mesenchymal stromal cells (UCMSCs). (**A**) Cell surface marker analyses revealed that the UCMSCs have a positive expression of CD44(+), CD73(+), CD90(+), and CD105(+) and negative expression of CD34(−) and CD11b(−), indicating that the UCMSCs expressed the definitive markers of MSCs. The mean intensity of CD105 expression on UCMSCs is lower. This may be related to a lower amount of CD105 expression on the cell surface of USMSCs, or the antibody directed against CD105 we used might possess less avidity of binding or fluorescein conjugation. (**B**) Western blot analyses confirmed the protein expression of CD9, CD63, CD81, and HSP70 in UCMSC exosomes but not in the drop-through fraction. (**C**) The number and size of UCMSC exosomes were 4.9 × 10^10^/mL and 151 nm before concentration; after 200-fold concentration, the protein concentration was 1.13 ± 0.03 mg/mL (2.2–7.8 × 10^12^ vesicles/mL). (**D**) RNA-seq analyses revealed the relative abundance of miRNAs in the UCMSC exosomes.

After UCMSCs were isolated from Wharton’s jelly of umbilical cord tissue, we used flow cytometry to examine MSC characteristics, such as specific cell surface markers and differentiation capacity. Our results revealed that UCMSCs were CD44(+), CD73(+), CD90(+), and CD105(+) (Figure 1A). Furthermore, CD34 and CD11b were not detected in the UCMSCs (Figure 1A).

### 2.2. Characterization (Size, Number, and Content) of UCMSC Exosomes

According to the guidelines of the Minimal Information for Studies of Extracellular Vesicles 2018 (MISEV2018) and the Multimodal Testing of Quality Control Parameters of UCMSC exosomes in the Vesicular Secretome Fraction of UCMSC exosomes [34], we prepared UCMSCs with a cell viability of more than 95%, and the cell surface markers of UCMSCs showed positive CD44, CD73, CD90, and CD105 expression and negative CD34 and CD11b expression (Figure 1A). To further characterize the UCMSC exosomes, we analyzed the tetraspanins, particle number, and size of UCMSC exosomes. Our data revealed that UCMSC exosomes were approximately 80–250 nm in size and 2–8 × 10^11^/mL in quantity, prepared from 4.5 × 10^7^ UCMSCs. The identities of exosomes were confirmed by the expression of CD63(+), CD81(+), CD9(+), and HSP70(+) (Figure 1B). The particle number of exosomes was 4.9 × 10^10^ vesicles/mL and 2.2–7.8 × 10^12^ vesicles/mL before and after concentration, respectively, with a protein concentration of 1.2 mg/mL and mean size of UCMSC exosomes of 151 nm (Figure 1C), which is known to somewhat overestimate the size of EVs [35]. The exosomes were identified as having >50% of CD81(+), as determined through flow cytometry in our previous study [36], which is higher than the requirement (10–15%) specified in the MISEV2018 guidelines [37]. The size of UCMSC exosomes was 151 nm, as detected using ZetaView NTA (Figure 1C).

Furthermore, RNA-seq analyses revealed the three most representative miRNAs in UCMSC exosomes to be the following: hsa-miR-125a-5p, hsa-miR-125b-5p, and hsa-miR-127-3p (Figure 1D). The 14 most abundant proteins detected in UCMSC exosomes (and relative abundance) were transforming growth factor-beta-induced ig-h3 (6.4%), collagen alpha-2(I) chain (4.7%), collagen alpha-1(I) chain (3.1%), glia-derived nexin (GDN, 2.1%), type IV collagenase (MMP2, 1.9%), actin (1.8%), fibronectin (1.7%), pentraxin-related protein PTX3 (1.3%), α-actinin-1 (1.1%), α-actinin-4 (1.0%), pyruvate kinase (PKM, 1.0%), complement C1s (1.0%), galectin-3-binding protein (1.0%), and sulfhydryl oxidase 1 (QSO × 1, 1.0%) (Table 1). Different growth factors in UCMSC exosomes were measured using multiplex bead immunoassay. The angiopoietin level was 173.5 ± 90.5 pg/mL, endoglin level was 87.3 ± 32.0 pg/mL, and VEGF-C level was 1353.0 ± 185.7 pg/mL. Taken together, we found that both structural proteins and functional macromolecules (e.g., miRNAs and growth factors) are present on and in the UCMSC exosomes and that different growth factors are present in UCMSC exosomes.

### 2.3. Protective Effects of UCMSC Exosomes on Cisplatin-Induced Hearing Loss in Mice

**Figure 2:**
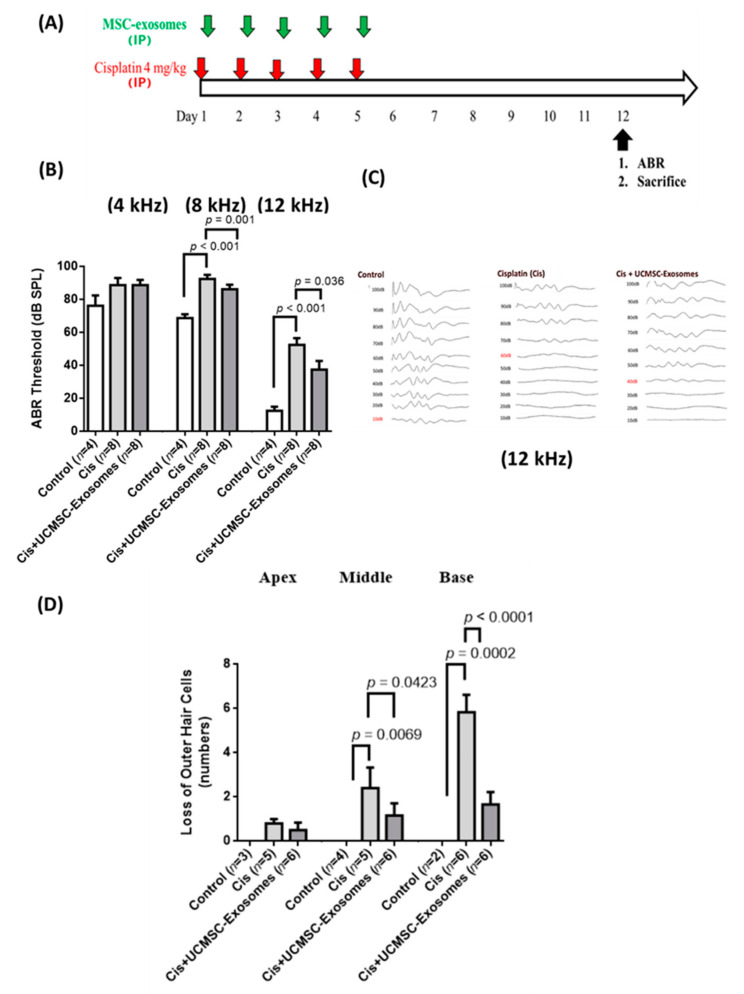
Effects of intraperitoneal injection of umbilical cord mesenchymal stromal cell (UCMSC) exosomes on cisplatin-induced hearing loss in mice. (**A**) Schematic of the experimental design of chronic administration of cisplatin in mice with induced hearing loss. Cisplatin (4 mg/kg) was injected intraperitoneally once per day for 5 consecutive days. UCMSC exosomes (1.2 μg/μL) in 100 μL of medium were administered intraperitoneally the same day after cisplatin injection. After 7 days, the hearing thresholds of all groups of mice were measured using an auditory brain response (ABR) instrument. Next, the mice were sacrificed to collect cochlear tissue for immunohistochemical staining. (**B**) ABR measurements revealed that UCMSC-exosome treatment significantly reduced the hearing thresholds at 8 and 12 kHz in the cisplatin-injected mice *n* = 4–8 per group. (**C**) The representative results of ABR at 12 kHz in each group of mice are shown. (**D**) Cisplatin-injected mice had significant loss of OHCs at the middle and basal regions compared with the control group. After UCMSC-exosome treatment in cisplatin-injected mice, the loss of OHCs at the middle and basal regions was significantly reduced compared with cisplatin-injected mice, *n* = 2–6 per group. Statistical significance was defined as *p* < 0.05.

**Figure 3:**
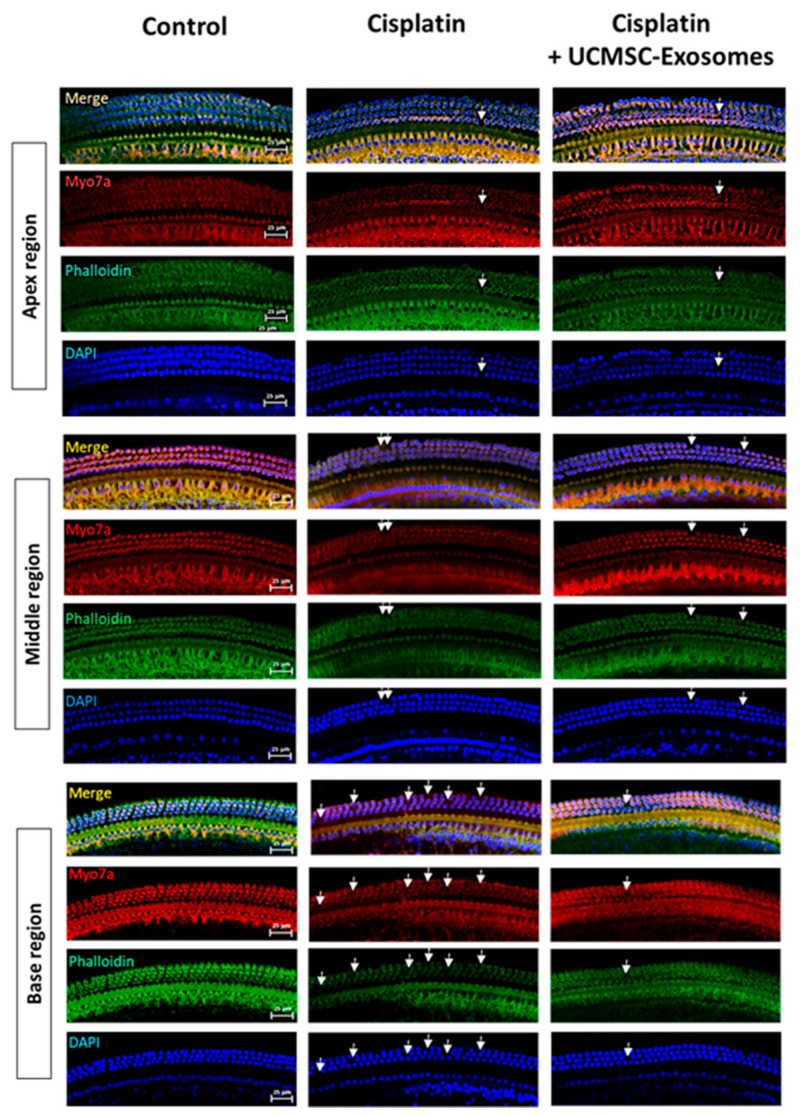
Immunohistochemical staining analyses. Representative results of immunohistochemical staining of the nucleus (DAPI, blue), hair cells (HCs, Myo7A, red), and cytoskeleton (phalloidin staining, green) at the regions of the apex, middle, and base of the cochlear tissues in each group of mice are shown. Scale bar = 25 μm. The loss of OHCs in the cochlear tissues is indicated by arrows.

In this study, we first investigated the protective effects of UCMSC exosomes on cisplatin-induced SNHL in mice. We injected cisplatin intraperitoneally at 4 mg/kg in the left abdomen of the mice once per day for 5 consecutive days (Figure 2A). Next, 1.2 mg/mL UCMSC exosomes were administered intraperitoneally in the right abdomen. Our data suggested that mice receiving chronic cisplatin injection exhibited a significant increase in hearing threshold (8 kHz: 92.50 ± 2.50 dB; 12 kHz: 52.50 ± 4.12 dB) compared with the control mice (8 kHz: 68.75 ± 2.39 dB; 12 kHz: 12.50 ± 2.50 dB, Figure 2B). Intraperitoneal UCMSC exosome injection in cisplatin-injected mice significantly reduced the hearing threshold of 8 and 12 kHz to 86.25 ± 2.80 dB and 37.50 ± 5.26 dB, respectively (Figure 2B,C). Moreover, OHC loss was examined immunohistochemically: anti-Myo7A and anti-phalloidin antibodies were used to investigate the locations and numbers of OHCs and inner HCs in the cochlear tissues. Our results revealed that cisplatin-injected mice exhibited significant loss of Myo7A(+) OHCs in the apex, middle, and base regions of the cochlear tissues (cisplatin: apex, 0.80 ± 0.20; middle, 2.40 ± 0.93; and base, 5.83 ± 0.79 vs. no loss in the control group; Figure 2D and Figure 3). Moreover, the treatment of UCMSC exosomes in cisplatin-injected mice significantly reduced the loss of Myo7A(+) OHCs in the middle and base regions of the cochlear tissues (cisplatin + UCMSC exosomes: apex, 0.50 ± 0.34; middle, 1.17 ± 0.54; and base, 1.67 ± 0.56; Figure 2D and Figure 3). Our results suggested that cisplatin-induced OHC loss was more significant in basal and middle regions than in the apex. Therefore, the rescue of OHCs by UCMSC exosomes was more significant in the basal and middle regions (Figure 2D).

### 2.4. The miRNA Expression Profiles of the Cochlear Tissues from Each Group of Mice

**Figure 4:**
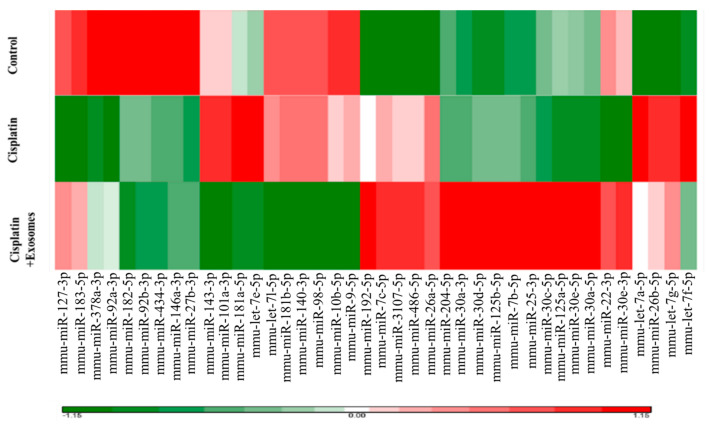
RNA-seq analyses for evaluating the effects of umbilical cord mesenchymal stromal cell (UCMSC) exosomes on the mouse miRNA expression patterns of the cochlear tissues of mice with cisplatin-induced hearing loss. Cochlear tissues were collected from a representative mouse in each group of mice according to the results for auditory brain response (ABR). Total RNA was extracted from the cochlear tissues and analyzed with RNA-seq. Notably, the treatment response of miRNAs (mmu-miR125b-5p, mmu-miR125a-5p, and mmu-miR127-3p) in inner ear tissues after UCMSC-exosome treatment overlapped with that of miRNAs present in UCMSC exosomes, including human miRNAs (125b-5p, 125a-5p, and 127-3p). This may be related to the direct uptake of homologous miRNAs of UCMSC exosomes by inner ear cells or the indirect induction of murine homology of miRNAs.

To investigate the underlying mechanisms of the protective effects of UCMSC exosomes on cisplatin-injected mice, we analyzed the miRNA expression profiles of representative cochlear tissues from each group of mice by using RNA sequencing technology. Our results revealed that cisplatin injection downregulated the expression of the following mmu-miRNAs in the cochlear tissues: mmu-miR-22-3p, mmu-miR-30e-3p, mmu-miR-27b-3p, mmu-miR-92a-3p, mmu-miR-92b-3p, mmu-miR-127-3p, mmu-miR-148a-3p, mmu-miR-182a-5p, mmu-miR-183-5p, mmu-miR-378a-3p, and mmu-miR-434-3p. By contrast, cisplatin injection upregulated the expression of the following miRNAs: mmu-miR26a-5p, mmu-miR-26b-5p, mmu-miR-101a-3p, mmu-miR3107-5p, mmu-miR-143-3p, mmu-miR-181a-5p, mmu-miR-192-5p, mmu-miR486-5p, mmu-let7a-5p, mmu-let7c-5p, mmu-let7e-5p, mmu-let7f-5p, and mmu-let7g-5p (Figure 4). Furthermore, UCMSC exosomes reversed the miRNA profile of the cochlear tissue from cisplatin-injected mice. Specifically, mmu-miR-22-3p, mmu-miR-25-3p, mmu-miR-30a-3p, mmu-miR-30a-5p, mmu-miR-30c-5p, mmu-miR-30 d-5p, mmu-miR-30e-5p, mmu-miR-30e-3p, mmu-miR-92a-3p, mmu-miR125a-5p, mmu-miR-125b-5p, mmu-miR-127-3p, mmu-miR-183-5p, mmu-miR-204-5p, mmu-miR-378a-3p, and mmu-let7b-5p were upregulated, and mmu-miR-9-5p, mmu-miR-10b-5p, mmu-miR-26b-5p, mmu-miR-98-5p, mmu-miR-101a-3p, mmu-miR-140-3p, mmu-miR-143-3p, mmu-miR-181a-5p, mmu-miR-181b-5p, mmu-let7a-5p, mmu-let7c-5p, mmu-let7e-5p, mmu-let7f-5p, and mmu-let7g-5p were downregulated (Figure 4). Notably, three human miRNAs—hsa-miR-125a-5p, hsa-miR-125b-5p, and hsa-miR-127-3p—were highly abundant in UCMSC exosomes (Figure 1D), implying that they regulate mouse miRNA expression in the cochlear tissues of cisplatin-injected mice.

### 2.5. Protective Effects of UCMSC Exosomes on Cisplatin-Induced Hearing Loss in Mice

**Figure 5:**
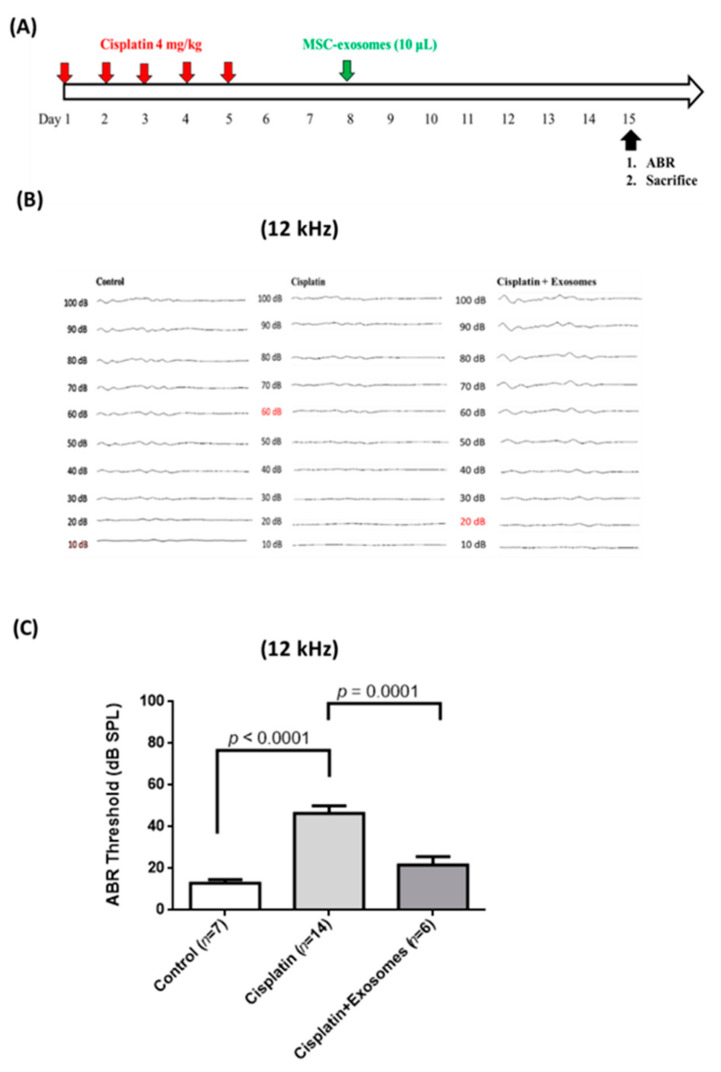
Effects of round window injection of umbilical cord mesenchymal stromal cell (UCMSC) exosomes on cisplatin-induced hearing loss in mice. (**A**) Schematic of the experimental design of chronic cisplatin administration for inducing hearing loss in mice. Cisplatin (4 mg/kg) was injected intraperitoneally once per day for 5 consecutive days, and 3 days later, UCMSC exosomes (1.2 mg/mL) in 10 μL of medium were administered through round window injection. After 7 days, the hearing thresholds of all groups of mice were measured using an auditory brain response (ABR) instrument at 12 kHz. Next, the mice were killed to collect cochlear tissue for immunohistochemical staining. (**B**) The results of ABR measurements revealed that cisplatin-injected mice had significantly impaired hearing function at 12 kHz compared with the control group. After UCMSC-exosome treatment in the cisplatin-injected mice, the hearing threshold at 12 kHz was significantly reduced compared with that of the cisplatin-injected mice, *n* = 6–14 per group. (**C**) Representative results of ABR at 12 kHz in each group of mice are shown. Statistical significance was defined as *p* < 0.05.

To further evaluate the protective effects of UCMSC exosomes on cisplatin-induced SNHL in mice, we designed another experiment to evaluate the post-treatment effect of UCMSC exosomes through RWN injection 3 days after cisplatin injection (Figure 5A). Cisplatin-injected mice exhibited a significant increase in hearing threshold (12 kHz: 46.43 ± 3.57 dB) compared with the control mice (12 kHz: 12.86 ± 1.84 dB, Figure 5B,C). RWN injection of UCMSC exosomes in cisplatin-injected mice significantly reduced the hearing threshold of 12 kHz (21.67 ± 4.01 dB) (Figure 5B,C).

### 2.6. Impairments of Biological Pathways in Mouse Cochlear Tissues after Cisplatin and UCMSC-Exosome RWN Injection

**Figure 6:**
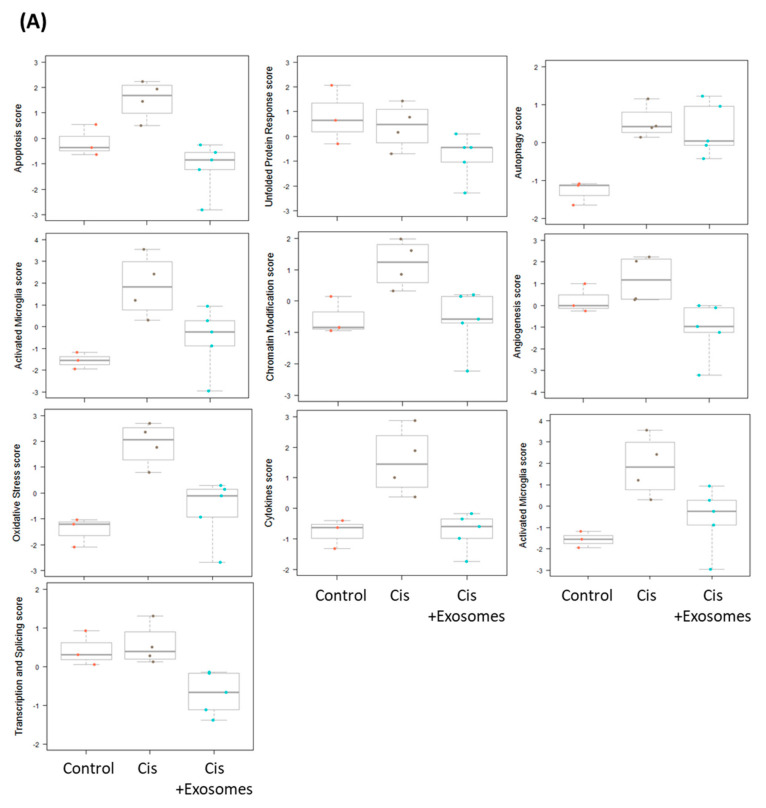

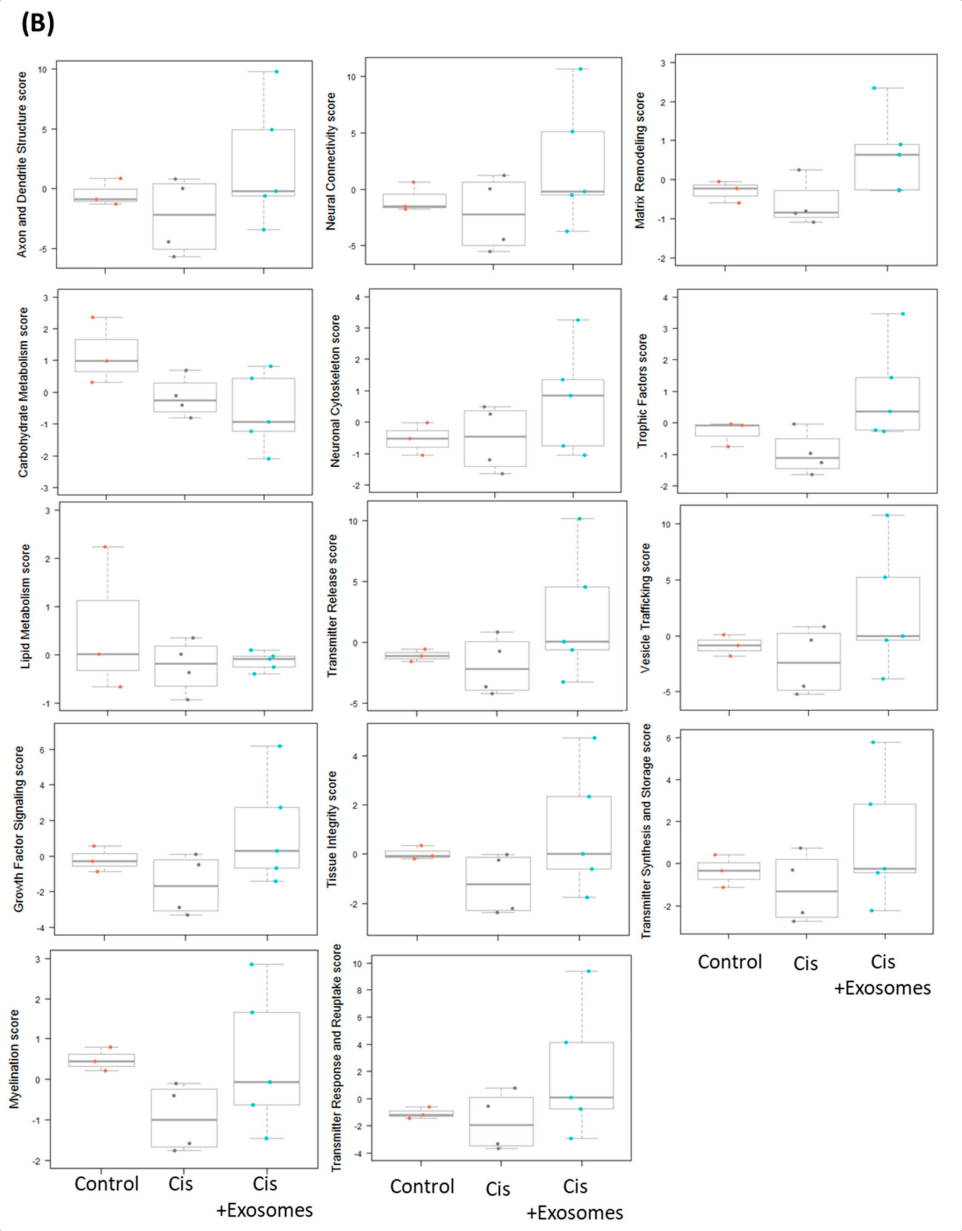

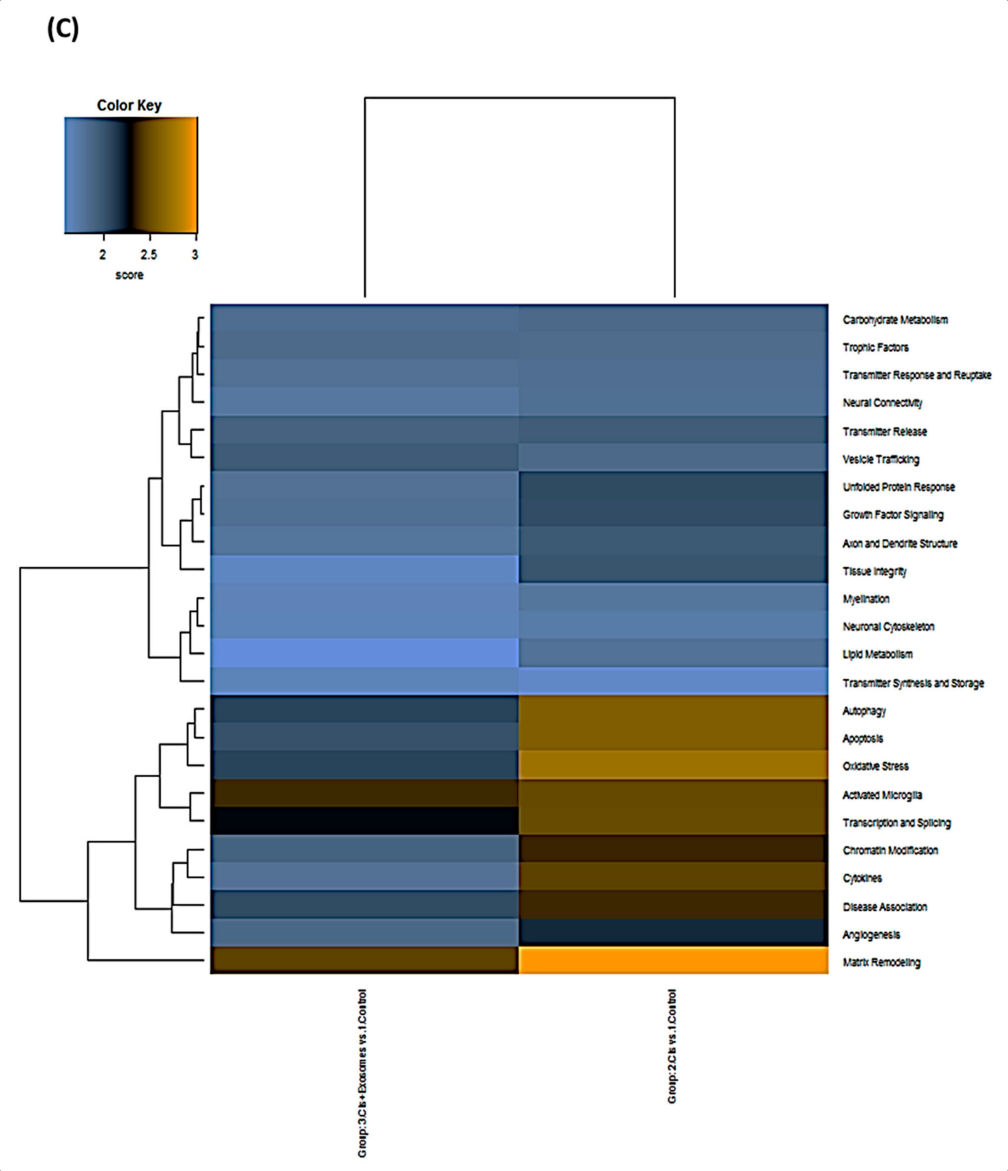

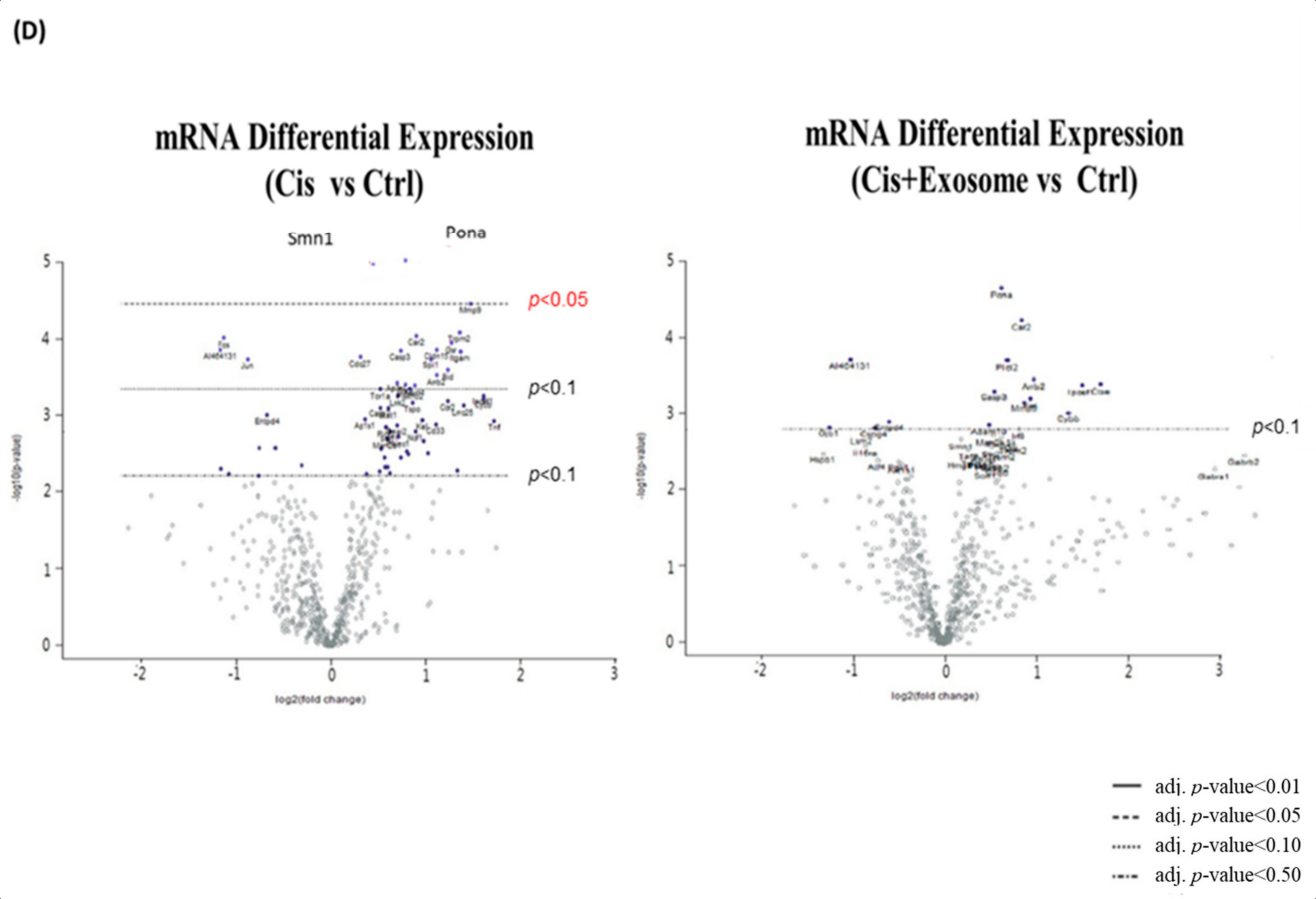
Neuropathological gene panel analyses of the biological pathways of umbilical cord mesenchymal stromal cell (UCMSC) exosomes in mice with cisplatin-induced hearing loss. (**A**) Increases in the gene scores of biological pathways in the mRNA expression profiles of the cochlear tissues were observed in three to five representative mice receiving chronic cisplatin injection. Ctrl = orange, Cis = gray, and Cis + Exosomes = blue. (**B**) Decreases in the gene scores involved the biological pathways in the mRNA expression profiles of the cochlear tissues in the representative mice receiving chronic cisplatin injection. Ctrl = orange, Cis = gray, and Cis + Exosomes = blue. (**C**) After normalization with the control group, the heatmap of gene expression profiles in each biological pathway of cisplatin vs. control (ctrl) and UCMSC exosomes (cisplatin mice treated with 1.2 mg/mL UCMSC exosomes) vs. control. The color codes represent the scores after normalization: positive scores are coded in brown or orange, and negative scores are coded in dark or light blue. (**D**) Volcano analyses indicated the differentially increased and decreased genes after normalization with ctrl mice. (i) SMN1 and Pona were significantly increased in the cisplatin group compared with in the control group. (ii) After UCMSC-exosome treatment, SMN1 was totally inhibited, and Pona was significantly reduced, since Pona was not significantly elevated (only above the line of adjusted *p*-value < 0.5 and does not reach the line of adjusted *p*-value < 0.1 and adjusted *p*-value < 0.05) in the cochlear tissues of cisplatin-injected mice. Statistical significance was defined as *p* < 0.05 in red color.

From our results of the ABR experiments in each group, we selected representative samples from the control (*n* = 3; the hearing threshold of three mice at 12 kHz was 10, 10, and 20 dB), cisplatin (n = 4; 30, 40, 40, and 60 dB), and cisplatin + UCMSC exosomes (*n* = 5; 20, 20, 10, 20, and 20 dB) groups for NanoString gene expression analyses. Total RNA was isolated from the representative cochlear tissues of each group. A NanoString neuropathology panel was adopted to analyze the biological significance of UCMSC-exosome treatment. In this analysis, many genes involved in critical neuropathological pathways were clustered together to monitor the damaging effects of cisplatin on cochlear tissues and the beneficial effects of UCMSC exosomes on damaged cochlea (Figure 6A,B). Our data suggested that the upregulated gene scores in the cochlear tissue of mice receiving chronic cisplatin injection were apoptosis, autophagy, chromatin modification, oxidative stress, activated microglia, angiogenesis, cytokines, transcription, and splicing, all of which were reversed after treatment with UCMSC exosomes (Figure 6C). Furthermore, the downregulated gene scores in the cochlear tissue of cisplatin-injected mice were axon and dendrite structure, carbohydrate metabolism, lipid metabolism, growth factor signaling, myelination, neural transmitter release, neural transmitter response and reuptake, neural transmitter synthesis and storage, trophic factors, and vesicle trafficking. The downregulation of most of these gene scores was effectively reversed after treatment with UCMSC exosomes, namely those of axon and dendrite structure, growth factor signaling, myelination, neural transmitter release, neural transmitter response and reuptake, neural transmitter synthesis and storage, trophic factors, and vesicle trafficking (Figure 5C). Volcano analyses further revealed that the significantly increased gene expression levels (mRNA levels) in the cisplatin group compared with the control group were SMN1 and Pona (Figure 6D, left panel), both of which were reduced after treatment with UCMSC exosomes (Figure 6D, right panel). Specifically, the mRNA levels of SMN1 and Pona were only above the line of adjusted *p*-value < 0.5 and do not reach the line of the adjusted *p*-value < 0.1 and adjusted *p*-value < 0.05, indicating that both SMN1 and Pona were not significantly elevated in the cisplatin + exosome group when compared with the control group (Figure 6D, right panel). Therefore, the results suggested that UCMSC-exosome treatment may reduce the upregulated mRNA levels of SMN1 and Pona.

### 2.7. Relative Abundance of Different Cell Types in Mouse Cochlear Tissues after Chronic Cisplatin Injection and UCMSC-Exosome Treatment through RWN Injection

**Figure 7:**
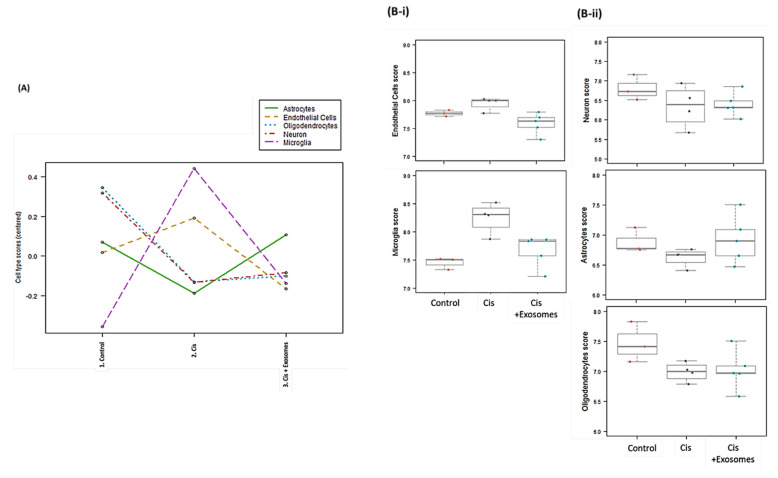
Neuropathological gene panel analyses of the involved cell types in the cochlear tissues from mice receiving chronic cisplatin injection. (**A**) Summary of the scores of cell types in the mRNA expression profiles of the cochlear tissues in three representative mice per group. (**B**) Increases and decreases in those scores.

Through neuropathological gene expression panel analyses, mRNA expression levels of typical cell marker genes of neurons, astrocytes, oligodendrocytes, endothelial cells, and microglial cells were clustered together to evaluate the damaging effects of chronic cisplatin administration on cochlear tissues and the beneficial effects of UCMSC exosomes on damaged cochlea (Figure 7A). Our data indicated that increased marker gene expression of microglia and endothelial cells was shown in the cochlear tissue of cisplatin-injected mice. UCMSC-exosome treatment reduced the increased gene scores of astrocytes, microglial cells, and endothelial cells (Figure 7B-i). By contrast, decreased marker gene expression of neurons, astrocytes, and oligodendrocytes was found in the cochlear tissue of mice receiving chronic cisplatin injection, and UCMSC-exosome treatment increased the gene scores of neurons but not of astrocytes or oligodendrocytes (Figure 7B-ii).

## 3. Discussion

We designed two independent experiments to evaluate the protective effects of UCMSC exosomes on cisplatin-induced hearing loss in mice. Our results revealed that the systemic administration of UCMSC exosomes improved the hearing of cisplatin-injected mice and protected cochlear OHCs from apoptosis and that intraperitoneal administration of UCMSC exosomes reversed the mouse mmu-miRNA expression pattern. Notably, the treatment response of miRNAs, including mmu-miR125b-5p, mmu-miR125a-5p, and mmu-miR127-3p, in inner ear tissues after UCMSC-exosome treatment overlapped with that of miRNAs present in UCMSC exosomes, including human miRNAs (125b-5p, 125a-5p, and 127-3p). This may be related to the direct uptake of homologous miRNAs of UCMSC exosomes by inner ear cells or the indirect induction of murine homology of miRNA expression. Human amniotic fluid-derived MSCs also abundantly express hsa-miR-125a to maintain their MSC properties [38]. Furthermore, hsa-miR-125b negatively regulates the osteogenic differentiation of periodontal ligament stem cells by targeting Cx43 [39]. Notably, hsa-miR-125b-5p is highly enriched in the EVs of bone marrow MSCs cultured in hypoxic conditions and facilitates ischemic cardiac repair by decreasing cardiomyocyte apoptosis [40]. Additionally, miR127-3p negatively regulates epithelial–mesenchymal transition [41]. Thus, the abundance of hsa-miR-125a-5p, hsa-miR-125b-5p, and hsa-miR-127-3p in the UCMSC exosomes may account for the effects on the protection of OHCs from death. Indeed, cisplatin-downregulated miRNAs, such as mmu-miR-182a-5p and mmu-miR-183-5p, were demonstrated to be involved in the maintenance of cochlear HC functions [42]. We will further clarify how these miRNAs are directly involved in the protection of OHCs in the future.

Proteomic analysis revealed that GDN and extracellular matrix (ECM) remodeling mediators, such as transforming growth factor beta-induced ig-h3 [43], collagen alpha-2(I) chain, collagen alpha-1(I) chain, MMP2, and fibronectin, are highly abundant in the UCMSC exosomes (Table 1). GDN is a serine protease inhibitor and can promote neurite outgrowth in the central nervous and olfactory systems [44,45]. GDN can regulate tissue remodeling by maintaining a balance with thrombin in the nervous system [46]. GDN has also been identified in the secretome of MSCs, which can improve the motor phenotype and neuronal properties of 6-hydroxydopamine-induced Parkinson’s disease in rats [47]. Additionally, MMP2, MMP9, and other ECM molecules contribute to cochlear tissue remodeling and blood–labyrinth barrier permeability in noise-induced hearing loss [48,49]. Our data indicated that UCMSC exosomes contain high amounts of galectin-3 and certain growth factors. Whether the protection of OHCs is related to galectin-3 or growth factors involved in tissue remodeling and integrity in cochlear tissues warrants further research. The expression of CD63 was much higher than the expression of CD9 and CD81 in UCMSC exosomes because exosomes derived from different lineages of cells differ in their expression of exosome markers [50]. In our study, proteomic analysis revealed that UCMSC exosomes contain more than 1500 proteins, some with high abundance and others with low abundance. Figure 1B indicates the different concentrations of tetraspanins identified. In the future, we will analyze whether differences in the expression of CD63, CD9, and CD81 influence the biological functions of UCMSC exosomes. Furthermore, the sizes and vesicles of UCMSC exosomes were evaluated using ZetaView NTA. The size of UCMSC exosomes ranged between 80 and 250 nm, which is larger than that (approximately 100 nm) measured using transmission electromicroscopy (TEM) in our previous study [36]. However, NTA devices are known to overestimate exosome size compared with TEM, and the sizes measured using ZetaView NTA were larger than those measured using NanoSight NS300 [36]. The mean vesicle size was 151 nm, and the mode size was 107 nm. Figure 1C reveals a distribution shoulder at 250 nm for the size of exosomes. This may be related to the heterogeneity of UCMSC exosomes.

Actin and several actin-associated proteins are abundant in exosomes and play a vital role in the polymerization machinery that stabilizes or depolymerizes microfilaments; they are also involved in the functions of integrins [51]. Proteomic analysis of LC/MS/MS after membrane solubilization and trypsin digestion for peptide identification demonstrated that UCMSC exosomes contain more than 1500 proteins matched by peptides, including both structural and functional proteins. For instance, we found that actin, alpha-actinin 1, and fibronectin were abundant structural proteins and may be linked to the kinesis and dynamics of exosomes. By contrast, secretory proteins, TGF-β and galectin-3, were also some of the 15 most abundant proteins and may be implicated in the repair of cisplatin-induced hearing loss. Other abundant proteins include MMP2, a matrix enzyme, and fibronectin, which may also be involved in tissue repair. We also measured cytokines and growth factors using multiplex bead assay and showed that FGF2 and VEGF were highly expressed in UCMSC exosomes. Together, these proteins and growth factors in UCMSC exosomes may participate in the repair of cisplatin-induced hearing loss. Further research is warranted to clarify our results and elucidate the mechanisms. In this small animal study, we simply used a two batches of tissue cultures at 300 milliliters for the harvest of exosomes derived from UCMSCs. After this pre-clinical study, we will proceed to clinical studies, which require a large amount of exosomes for clinical administration. We need to expand the 2D culture system to a 3D culture system by which a large amount of exosomes could be harvested by an automated hallow fiber filtration system.

In the present study, the local administration of UCMSC exosomes through RWN injection more effectively rescued the hearing loss of cisplatin-injected mice than intraperitoneal injection (Figure 5). Nanostring neuropathological gene expression panels have been widely used to study neuropathological changes through analysis of the relevant mRNA levels of each biological pathway [52,53]. We used it to clarify the underlying mechanism of the protective effects of UCMSC exosomes on chronic cisplatin-induced hearing loss in mice (Figure 6 and Figure 7). Notably, apoptosis, autophagy, chromatin modification, oxidative stress, activated microglia, angiogenesis, cytokine score, and transcription and splicing score were upregulated in the cochlear tissues of mice receiving chronic cisplatin injection (Figure 6A). Moreover, gene analytic scores in the biological processes of chromatin modification, oxidative stress, activated microglia, angiogenesis, cytokines, transcription, and splicing were involved in the ototoxicity of chronic cisplatin administration. The downregulated biological processes in the cochlear tissue of cisplatin-injected mice were matrix remodeling, tissue integrity, axon and dendrite structure, growth factor signaling, myelination, neural connectivity, neuronal skeleton, neural transmitter release, neural transmitter response and reuptake, neural transmitter synthesis and storage, trophic factors, and vesicle trafficking (Figure 6B). After normalization with the control group, our findings imply that UCMSC-exosome treatment can reverse most biological abnormalities in the cochlear tissues of mice receiving chronic cisplatin injection (Figure 6C). Intravenous injection of human UC blood MSCs can significantly improve hearing loss by increasing the number of SGNs [10].

Notably, our data revealed that activated microglia and endothelial cells were upregulated in the cochlear tissues of mice receiving chronic cisplatin injection (Figure 7). Microglia may be activated in the cochlear nucleus and may contribute to tissue remodeling in hearing loss [54]. In noise-induced hearing loss, microglial activation was detected in cochlear tissue within 10 days after noise exposure, reaching the highest level at 30 days [55]. In the present study, UCMSC-exosome treatment was found to effectively reverse the abnormal changes in gene expression scores in endothelial cells, activated microglia, and astrocytes in the cochlear tissues of cisplatin-injected mice (Figure 6).

## 4. Materials and Methods

### 4.1. Source and Preparation of UCMSCs

The use of UC tissue and processing for the isolation of UCMSCs was approved by the Institutional Review Board of Mackay Memorial Hospital, Taipei, Taiwan (15MMHIS106). UCMSCs were derived from the UC tissues. UCs were washed with PBS under a sterile laminar flow cell culture hood and cut into 5 cm^2^ segments. The segments were cut longitudinally, blood vessels were removed, and segments were transferred to 25 cm^2^ flasks. The culture of UCMSCs was in adhesion condition and initially fed with 2500 cells/cm^2^ in a 12-well plate with low-glucose DMEM (Gibco; Thermo Fisher Scientific, Inc., Waltham, MA, USA) with 10% FBS at 37 °C in humid air with 5% CO_2_. The culture medium was changed every 3 days. The UCMSCs prepared from this procedure can differentiate into an osteogenic lineage, as shown in our previous report [56], in which we demonstrated that they are comparable to bone marrow-derived MSCs in growth and differentiation related to genetic and proteomic modifications. UCMSCs between 3 and 8 passages were prepared and used for this study, as described previously [36,56]. We prepared the UCMSCs with cell viability over 95% and assessed them as follows to confirm the MSC characteristics (see Supplementary materials).

### 4.2. Flow Cytometric Analyses of UCMSC Surface Markers

UCMSC surface markers were detected with a Cytoflex flow cytometer (Beckman), which is a multicolor instrument with three lasers—blue (488 nm), red (633 nm), and violet (405 nm), the data readouts from which were processed using Cytoflex CytExpert Acquisition and Analysis Software Version 2.4 in accordance with the manufacturer’s instructions. Before the flow cytometric analyses, cells (2 × 10^5^ cells, UCMSCs at 2 × 10^6^ cells/mL) were suspended in 0.1 mL of phosphate-buffered saline (PBS) and incubated at room temperature for 30 min with the first antibodies (mouse antihuman) for immunostaining of UCMSCs, including CD44 PE (550989, BD), CD73 APC (560847, BD), CD90 FITC (555595, BD), CD105 PerCP-Cy5.5 (560819, BD), CD34 FITC (560942, BD), and CD11b PE (561795, BD), in comparison with internal control antibodies, including PE (556656, BD), APC (555751, BD), FITC (555748, BD), and PerCP-Cy5.5 (562438, BD). After direct immunostaining, the cells were washed twice in PBS buffer before analysis with Cytoflex Software.

### 4.3. Preparation of UCMSC Exosomes

**Figure 8:**
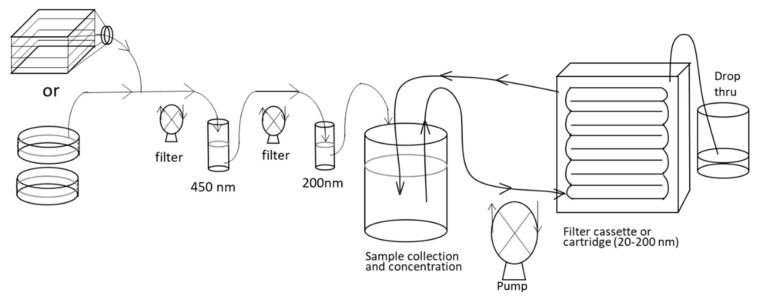
Isolation of UCMSC exosomes.

To isolate UCMSC exosomes, the UCMSC culture was initially fed with 4 × 10^4^ cells/mL in 10 mL of low-glucose DMEM with 10% FBS. When the cells reached 80% confluency (1.5 × 10^5^ cells/mL), the culture medium was washed and replaced with serum-free low-glucose DMEM for 48 h before exosomes were harvested. The serum-free medium was used to eliminate the contamination of the culture medium with 10% FBS and stimulate the release of exosomes by UCMSCs. In each experiment, we cultured and harvested 30 culture dishes (4.5 × 10^7^ cells), and the supernatant (300 mL) was collected for a series of filtrations: a 450-nm filter was used to filter out cell debris, followed by a 200-nm filter for filtering out apoptotic bodies and finally a cassette (cartridge) for retaining exosomes between 20 and 200 nm. (Figure 8).

### 4.4. Nanoparticle Tracking Analysis (NTA)

The size distribution of UCMSC exosomes was analyzed using nanoparticle tracking analysis (NTA) (ZetaView; Particle Metrix, Meerbusch, Germany) equipped with an sCMOS camera. NTA is used to visualize and analyze particles in liquids based on Brownian motion of particle size and viscosity and the temperature of the liquid. The UCMSC exosomes harvested from 300 mL of culture were diluted in 1 mL of PBS buffer at a 1:100 ratio, prepared through filtration using a 0.22 µm filter, and autoclaved before use. Each sample measurement was performed in three cycles through the scanning of 11 cell positions in which each scan captured 60 frames per position under the following settings: autofocus; camera sensitivity, 92.0; scattering intensity, 4.0; and temperature, 25 °C. After scanning, the videos were analyzed with the built-in ZetaView v8.05.05 SP2 software with the following parameters: particle size between 30 and 1000 nm and an embedded 40 mW laser with a wavelength of 488 nm. In a series of preparations, we measured the quantities of exosomes between 2 × 10^11^ and 8 × 10^11^/mL, which were prepared from 4.5 × 10^7^ UCMSCs. We pooled two batches of UCMSC exosomes (each batch with 300 mL of supernatant was concentrated into 1.5 mL). The pooled exosomes (3.0 mL) were aliquoted into five vials at 0.6 mL per aliquot and stored at −80 °C until analysis. Each vial was used within 1 day after thawing without reuse, thus avoiding another freeze/thaw cycle.

### 4.5. Western Blot Analysis of UCMSC-Exosome Markers

We used Western blot analysis to confirm the identification of UCMSC exosomes, including CD9, CD63, CD81, and HSP70 expression. The UCMSC exosomes (1.2 mg/mL) were lysed using a RIPA buffer containing 0.2 mg/mL PMSF protease inhibitor (Roche, Basel, Switzerland). The protein samples (20 μg) derived from UCMSC exosomes were loaded onto 12.5% polyacrylamide gel electrophoresis (SDS-PAGE). Then, the separated protein gels were transferred onto a PVDF membrane (Bio-Rad, cat no. 1620177) followed by blocking with 5% nonfat dry milk in Tris-buffered saline with Tween 20 (0.2%) for 30 min. The membranes were next incubated with primary antibodies: CD63 (clone EPR5702, ab134045, Abcam), CD81 (clone 1D6, GTX43505, GeneTex), CD9 (D8O1A,13174s, Cell Signaling Technology), or HSP70 (4872, Cell Signaling Technology) in 5% blotting grade Blocker BSA in TBS-Tween overnight at 4 °C. The membrane was subsequently washed three times before incubation with secondary antibodies using goat antimouse IgG (AP124P) at a 1:1000 dilution in 5% blotting buffer of nonfat dry milk for 30 min. Finally, the membranes were analyzed with an ECL T-Pro LumiLong Plus Chemiluminescence Detection kit (No. JT96-K004M).

### 4.6. Liquid Chromatography and Tandem Mass Spectrometry/Mass Spectrometry

After confirmation of the UCMSC exosomal protein markers, the protein samples were analyzed through liquid chromatography and tandem mass spectrometry/mass spectrometry (LC-MS/MS). The protein samples (200 μg) were trypsinized into peptides, evaporated until dry in a vacuum, and centrifuged before high-performance liquid chromatography was performed on a capillary column (C18 110A; 150 × 2.00-mm Phenomenex) at a flow rate of 0.2 mL/min in the reversed-phase mode using H_2_O and 80% acetonitrile (ACN) as mobile phases A and B, respectively. All procedures for proteomic analyses were conducted by Biotools, Taiwan.

### 4.7. Protein Concentration and Multiplex Cytokine Assays of UCMSC Exosomes

The protein concentration measured using a bicinchoninic acid assay kit (Thermo Fisher Scientific) in different batches was 1.13 ± 0.03 mg/mL after concentration. Different growth factors in UCMSC exosomes were measured using multiplex bead immunoassay (xMAP^®^, Merck KGaA, Darmstadt, Germany).

### 4.8. Animals

The protocols for the care and use of animals were approved by the Animal Care Committee of Mackay Medical College (A1060024). Eight-week-old C57BL/6 mice were purchased from BioLasco (Taiwan). In brief, ototoxicity was induced through the intraperitoneal injection of cisplatin, and the disease model was established as described by [57]. In our previous report, we demonstrated that intrathecal infusion of 20 μL of UCMSC exosomes at a concentration of 1.2 μg/μL can significantly reduce nerve injury-induced pain in rats compared with the concentrations of 0.12 and 0.6 μg/μL [36]. We accordingly chose the UCMSC exosomes at 1.2 μg/μL for intraperitoneal injection. We injected 100 μL of UCMSC exosomes intraperitoneally and 10 μL of UCMSC exosomes through the round window niche (RWN). For the intraperitoneal injection, 100 μL of UCMSC exosomes or serum-free medium was injected into the mice on the same day after cisplatin injection (Figure 2A). For the RWN injection, approximately 10 μL of UCMSC exosomes or medium was injected into the mice through RWN injection 3 days after cisplatin administration (Figure 5A). For both injection routes, all mice were euthanized 1 week after injection to measure their auditory brainstem response (ABR) (Biopac Systems, Inc., Goleta, CA, United States). ABR was measured to detect the hearing threshold in mice [10,58,59]. In brief, the ABR data were recorded from the scalp of the mice using subdermal needle electrodes. The electrodes were placed in three places: (1) the vertex, (2) below the pinna of the left ear, and (3) below the contralateral ear (ground). The stimuli included clicks (100 ms duration; 4, 8, and 12 kHz), and ABR measurements were obtained with small animal ABR instruments (BIOPAC Systems). Acoustic stimuli were introduced directly into the ear canal. The sound stimulus intensities used in this study ranged from 10 to 100 dB. The ABR thresholds were measured through detection of the presence of wave V. After measurement of ABR, cochlear tissues were collected for immunohistochemical staining and gene expression analyses.

### 4.9. Whole-Mount Dissection, Confocal Microscopy, and Outer Hair Cell Counts

Cochlear tissues were isolated and dissected. Whole-mount dissections were performed as described by [60]. In brief, the excess temporal bone around the cochlea was trimmed. The cochlear tissue was bisected, submerged in PBS solution, and fixed with 4% paraformaldehyde. Subsequently, the cochlea was decalcified for 1 h. The primary antibodies were anti-Myo7a (1:100, Santa Cruz), which was incubated at 4 °C overnight, and antiphalloidin conjugated with Alexa 488 (1:200, Thermo), which was incubated at 4 °C for 2 h. The secondary antibody was rhodamine goat antimouse (1:200, KPL), which was incubated at room temperature for 2 h. The tissues were stained with DAPI (Sigma) and imaged using a confocal spectral microscope (Leica). Next, outer hair cells (OHCs) were counted on the basis of Myo7a and DAPI staining. Intact, Myo7a and DAPI-stained OHCs were counted directly when visible. In the whole-mount staining experiments, one cochlear tissue from the leaf ear of the mouse was placed on the slide for whole-mount staining. For the loss of OHCs counts, HCs were counterstained with phalloidin, Myo7a, and DAPI stainings. Images were captured using a confocal spectral microscope (Leica SP8). We acquired images at three randomly selected regions (300 μm length) in the apical, middle, and basal cochlear turns by using the same apparatus, and we manually counted the loss of OHCs. Then, the average loss of OHCs were calculated in the apex, middle, and base regions, respectively [61]. The number of mice used in the experiments is shown in Figure 2B,D. Since the cochlear tissues from some mice were not successfully collected, the number of cochlear tissues in Figure 2D is less than the number of mice in Figure 2B. OHC counts were compared using one-way ANOVA. *p* < 0.05 was considered to indicate statistical significance.

### 4.10. RNA Sequencing Analyses for miRNA Profiles in UCMSC Exosomes

The RNA samples in UCMSC exosomes were harvested with QIAzol Lysis Reagent (QIAGEN, Germany), 700 μL of which was added to EV pellets (1.2 mg/mL) with gentle vortexing for 5 min; next, 140 μL of chloroform was added, followed by vigorous shaking for 15 s and separation. The RNA samples were harvested by 12,000× *g* at 4 °C for 15 min in accordance with the manufacturer’s instructions. The small RNAs in the samples were subjected to RNA sequencing (RNA-seq) using next-generation sequencing (NGS). The RNA samples collected from UCMSC exosomes were prepared with TruSeq^®^ Small RNA (Illumina). The RNA fragments were ligated with 5′ and 3′ adaptors at both ends, resulting in approximately 140-bp cDNA products in gel electrophoresis for the detection of cDNA products. The small RNA samples were subjected to NGS analysis of miRNA profiles by Genomics (Taiwan). We performed RNA-seq analysis for the UCMSC exosomes and validated the miRNA profiles by RT-PCR in UCMSC exosomes. Our data suggested a reproducible and comparative expression of five miRNAs (191-5p, 125b-5p, Let-7f-5p, 125a-5p, and 127-3p) in five replicate samples. These miRNAs are commonly found in MSC-derived exosomes [62]. Notably, the treatment response of miRNAs, including mmu-miR125b-5p, mmu-miR125a-5p, and mmu-miR127-3p, in inner ear tissues after UCMSC-exosome treatment overlapped with that of miRNAs extant in UCMSC exosomes, including human miRNAs (125b-5p, 125a-5p, and 127-3p). This may be related to the direct uptake of homologous miRNAs of UCMSC exosomes by inner ear cells or the indirect induction of murine homology of miRNA expression. The expression levels of miRNA profiles in Figure 1D were obtained through the normalization of the U6B small nuclear RNA as an internal control based on a comparison of Ct values. We washed and replaced the 10% FBS medium with a serum-free low-glucose DMEM for 48 h before harvesting the UCMSC exosomes. The use of the serum-free medium for the harvest of UCMSC exosomes could eliminate the contamination of the medium with 10% FBS and promote exosome release by UCMSCs.

### 4.11. Nanostring Gene Panel Analysis of the Cochlear Tissues of Mice Receiving Chronic Cisplatin Injection

Cochlear tissues were obtained from representative mice in each group for total mRNA extraction. Then, total mRNA was directly subjected to analysis using the NanoString mouse neuropathology panel (NanoString Technologies, Seattle, WA, USA). Gene expression values involved in the neuropathological processes were clustered and analyzed. NanoString gene expression and bioinformatics analyses were performed by Cold Spring Biotech, Taiwan. The concentration of RNA that can be extracted from each cochlear tissue sample is low; the advantage of the NanoString neuropathological gene expression panel is the direct analysis of mRNA, which requires an RNA concentration of only 25 ng, thus serving as a powerful systemic gene expression platform for cochlear tissue. The results of a pathway score or cell type-specific score analyses can be complementary to those obtained from one more focused on individual genes. A web program PLAGE (Pathway Level Analysis of Gene Expression) was used to perform the kinds of analyses described in the literature [63]. Gene or pathway scores are used to summarize the data from a pathway’s genes or cell type-specific genes into a single score by using nCounter^®^ Advanced Analysis Software (version 2.0.134; https://www.nanostring.com/products/analysis-software/advanced-analysis; accessed on 15 March 2020). These scores are calculated as the first principal component of the pathway genes’ normalized expression. All the analyses of gene or pathways scores were according to the user manual of nCounter^®^ Advanced Analysis Software.

### 4.12. Statistical Analyses

Data are presented as means ± SD. We used one-way ANOVA to compare the groups. PRISM 6.0 software was used to perform one-way ANOVA and post hoc test (Tukey’s multiple comparisons test). Statistical significance was defined as *p* < 0.05.

## 5. Conclusions

This study demonstrates the protective effects of UCMSC exosomes on cisplatin-induced hearing loss in mice. We previously reported that UCMSC exosomes could ameliorate traumatic neuropathic pain though their anti-inflammatory effects by reducing TNF-α and IL-1β expression, leading to higher expression of nerve growth factors, including brain-derived neurotrophic factors and glial cell-derived neurotrophic factors [36]. In this study, we demonstrated that UCMSC exosomes rescued cisplatin-induced hearing loss associated with an increase in GDN expression and mmu-miR-125a-5p, mmu-miR-125b-5p, and mmu-miR127-5p expression in inner ear tissues, which may suppress inflammation or protect OHCs from damage. We also found that UCMSC exosomes contain higher levels of fibronectin, galectin-3, and certain growth factors. On the basis of these data, we propose that the underlying mechanism for the protective effects of UCMSC exosomes on cisplatin-induced hearing loss in mice is mediated by the uptake of miRNAs and remodeling factors of UCMSC exosomes that induce the expression of murine protective miRNAs and growth factors for the protection or repair of OHCs and protection against hearing loss.

## Figures and Tables

**Table 1:** Proteomic analyses of mesenchymal stem cell exosomes.

Protein Identification	Score	Relative Abundance (%)
Transforming growth factor-beta-induced ig-h3 [BGH3_HUMAN]	14,332.76	6.4
Collagen alpha-2(I) chain [CO1A2_HUMAN]	7154.01	4.7
Collagen alpha-1(I) chain [CO1A1_HUMAN]	5548.62	3.1
Glia-derived nexin [GDN_HUMAN]	3787.54	2.1
72 kDa type IV collagenase [MMP2_HUMAN]	3067.72	1.9
Actin, cytoplasmic 2 [ACTG_HUMAN]	4345.01	1.8
Fibronectin [FINC_HUMAN]	2620.17	1.7
Pentraxin-related protein PTX3 [PTX3_HUMAN]	2183.74	1.3
Alpha-actinin-1 [ACTN1_HUMAN]	2372.41	1.1
Alpha-actinin-4 [ACTN4_HUMAN]	1758.24	1.0
Pyruvate kinase PKM [KPYM_HUMAN]	1960.58	1.0
Complement C1s subcomponent [C1S_HUMAN]	1353.73	1.0
Galectin-3-binding protein [LG3BP_HUMAN]	2128.30	1.0
Sulfhydryl oxidase 1 [QSOX1_HUMAN]	1251.85	1.0

## Data Availability

We will make data available as much as we can on a reasonable request.

## Supplementary Materials

The following are available online at https://www.mdpi.com/article/10.3390/ijms22136664/s1.
