# Supplementary material for: Umbilical Cord Mesenchymal Stromal Cell-Derived Exosomes Rescue the Loss of Outer Hair Cells and Repair Cochlear Damage in Cisplatin-Injected Mice
Source: Int J Mol Sci. 2021 Jun 22;22(13):6664. doi: 10.3390/ijms22136664 (PMC8267798; doi:10.3390/ijms22136664)
Supplement: Supplementary file 1 [file ijms-22-06664-s001.zip › ijms-1241910-supplementary.pdf]

Supplementary File

UCMSCs, isolation of exosomes and analysis of the protein profiles and growth factors

Source and preparation of UCMSCs

The use of UC tissue and processing for the isolation of UCMSCs was approved by the Institutional Review Board of Mackay Memorial Hospital, Taipei, Taiwan (15MMHIS106). The UCMSCs were cultured in low-glucose Dulbecco's modified Eagle's medium (DMEM) (Gibco; Thermo Fisher Scientific, Inc., Waltham, MA, USA) supplemented with 10% fetal bovine serum (FBS) (Gibco; Thermo Fisher Scientific, Inc., Waltham, MA, USA) at 37 °C in humid air with 5% CO_2. The culture of UCMSCs was initially fed with 4×10^4 cells/mL in 10 mL of low-glucose DMEM with 10% FBS. The culture medium was changed every 3 days. The UCMSCs prepared from this procedure can differentiate into an osteogenic lineage, as shown in our previous report [35], in which we demonstrated that they are comparable to bone marrow–derived MSCs in growth and differentiation related to genetic and proteomic modifications. UCMSCs between 3 and 8 passages were prepared and used for this study, as described previously [35, 36]. We prepared the UCMSCs with cell viability over 95% and assessed them as follows to confirm the MSC characteristics.

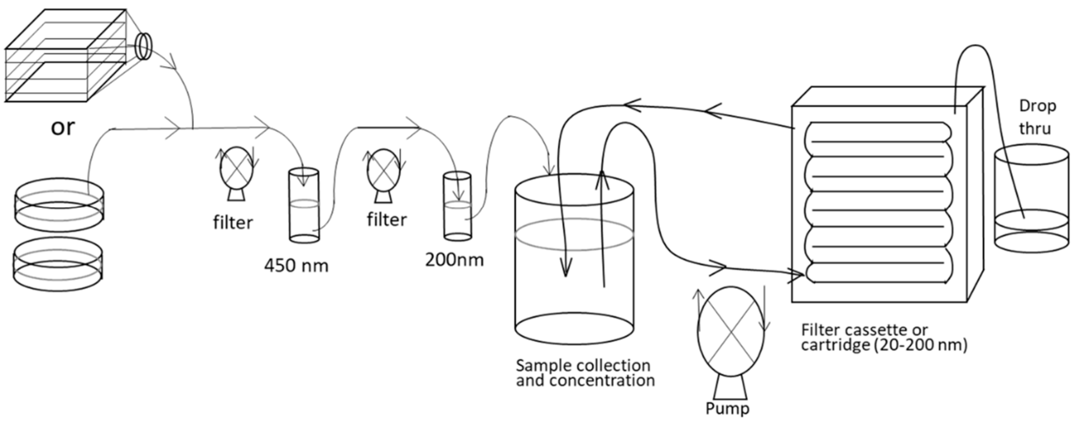

Figure S1: The way to harvest EVs.

Flow cytometric analyses of UCMSC surface markers

UCMSC surface markers were detected with a Cytoflex flow cytometer (Beckman), which is a multicolor instrument with three lasers—blue (488 nm), red (633 nm), and violet (405 nm)- data readouts from which were processed using Cytoflex Software in accordance with the manufacturer's instructions. Before the flow cytometric analyses, cells (2×10^5 cells, UCMSCs at 2×10^6 cells/mL) were suspended in 0.1 mL of phosphate-buffered saline (PBS) and incubated at room temperature for 30 min with the first antibodies (mouse antihuman) for immunostaining of UCMSCs, including CD44 PE (550989, BD), CD73 APC (560847, BD), CD90 FITC (555595, BD), CD105 PerCP-Cy5.5 (560819, BD), CD34 FITC (560942, BD), and CD11b PE (561795, BD), in comparison with internal control antibodies, including PE (556656, BD), APC (555751, BD), FITC (555748, BD), and PerCP-Cy5.5 (562438, BD). After direct immunostaining, the cells were washed twice in PBS buffer before analysis with Cytoflex Software.

Preparation of UCMSC exosomes

To isolate UCMSC exosomes, the UCMSC culture was initially fed with 4×10^4 cells/mL in 10 mL of low-glucose DMEM with 10% FBS. When the cells reached 80% confluency (1.5×10^5 cells/mL), the culture medium was washed and replaced with serum-free low-glucose DMEM for 48 h before exosomes were harvested. The serum-free medium was used to eliminate the contamination of the culture medium with 10% FBS and stimulate the release of exosomes by UCMSCs. In each experiment, we cultured and harvested 30 culture dishes (4.5×10^7 cells), and the supernatant (300 mL) was collected for a series of filtrations: a 450-nm filter was used to filter out cell debris, followed by a 200-nm filter for filtering out apoptotic bodies and finally a cassette (cartridge) for retaining exosomes between 20 and 200 nm (Supplementary Fig. 1).

Nanoparticle tracking analysis (NTA)

The size distribution of UCMSC exosomes was analyzed using nanoparticle tracking analysis (NTA) (ZetaView; Particle Metrix, Germany) equipped with an sCMOS camera. NTA is used to visualize and analyze particles in liquids based on Brownian motion of particle size and viscosity and the temperature of the liquid. The UCMSC exosomes harvested from 300 mL of culture were diluted in 1 mL of PBS buffer at a 1:100 ratio,

prepared through filtration using a 0.22-μm filter and autoclaved before use. Each sample measurement was performed in three cycles through the scanning of 11 cell positions in which each scan captured 60 frames per position under the following settings: autofocus; camera sensitivity, 92.0; scattering intensity, 4.0; and temperature, 25 °C. After scanning, the videos were analyzed with the built-in ZetaView v8.05.05 SP2 software with the following parameters: particle size between 30 and 1000 nm and an embedded 40 mW laser with a wavelength of 488 nm. In a series of preparations, we measured the quantities of exosomes between 2×10^{11} and 8×10^{11}/mL, prepared from 4.5×10^{7} UCMSCs. We pooled two batches of UCMSC exosomes (each batch with 300 mL of supernatant was concentrated into 1.5 mL). The pooled exosomes (3.0 mL) were aliquoted into five vials at 0.6 mL per aliquot and stored at −80 °C until analysis. Each vial was used within 1 day after thawing without reuse, thus avoiding another freeze/thaw cycle.

Western blot analysis of UCMSC-exosome markers

We used Western blot analysis to confirm the identification of UCMSC exosomes, including CD9, CD63, CD81, and HSP70 expression. The UCMSC exosomes (1.2 mg/mL) were lysed using a RIPA buffer containing 0.2 mg/mL PMSF protease inhibitor (Roche, Basel, Switzerland). The protein samples (20 μg) derived from UCMSC exosomes were loaded onto 12.5% polyacrylamide gel electrophoresis (SDS-PAGE). The separated protein gels were then transferred onto a PVDF membrane (Bio-Rad, cat no. 1620177) followed by blocking with

5% nonfat dry milk in Tris-buffered saline with Tween 20 (0.2%) for 30 min. The membranes were next incubated with primary antibodies: CD63 (clone EPR5702, ab134045, Abcam), CD81 (clone 1D6, GTX43505, GeneTex), CD9 (D8O1A,13174s, Cell Signaling Technology), or HSP70 (4872, Cell Signaling Technology) in 5% blotting grade Blocker BSA in TBS-Tween overnight at 4 °C. The membrane was subsequently washed three times before incubation with secondary antibodies using goat antimouse IgG (AP124P) at a 1:1000 dilution in 5% blotting buffer of nonfat dry milk for 30 min. Finally, the membranes were analyzed with an ECL T-Pro LumiLong Plus Chemiluminescence Detection kit (No. JT96-K004M).

Liquid chromatography and tandem mass spectrometry/mass spectrometry

After confirmation of the UCMSC exosomal protein markers, the protein samples were analyzed through liquid chromatography and tandem mass spectrometry/mass spectrometry (LC-MS/MS). The protein samples (200 μg) were trypsinized into peptides, evaporated until dry in a vacuum, and centrifuged before high-performance liquid chromatography was performed on a capillary column (C18 110A; 150 × 2.00-mm Phenomenex) at a flow rate of 0.2 mL/min in the reversed-phase mode using H_2O and 80% acetonitrile (ACN) as mobile phases A and B, respectively. All procedures for proteomic analyses were conducted by Biotools, Taiwan.

Protein concentration and multiplex cytokine assays of UCMSC exosomes

The protein concentration measured using a bicinchoninic acid assay kit (Thermo Fisher Scientific) in different batches was 1.13 ± 0.03 mg/mL after concentration. Different growth factors in UCMSC exosomes were measured using multiplex bead immunoassay (xMAP®, Merck KGaA, Germany).
